# Empiric therapy for hospital-acquired, Gram-negative complicated intra-abdominal infection and complicated urinary tract infections: a systematic literature review of current and emerging treatment options

**DOI:** 10.1186/s12879-015-1054-1

**Published:** 2015-08-05

**Authors:** Yoav Golan

**Affiliations:** Tufts Medical Center, Department of Medicine, Division of Geographic Medicine and Infectious Disease, 800 Washington St, Boston, MA 02446 USA

**Keywords:** Carbapenem, Piperacillin-tazobactam, Tigecycline, Ceftolozane-tazobactam, Ceftazidime-avibactam, Complicated intra-abdominal infection, Complicated urinary tract infection

## Abstract

**Background:**

Empiric therapy for healthcare-associated infections remains challenging, especially with the continued development of Gram-negative organisms producing extended-spectrum β-lactamases (ESBLs) and the threat of multi-drug–resistant organisms. Current treatment options for resistant Gram-negative infections include carbapenems, tigecycline, piperacillin-tazobactam, cefepime, ceftazidime, and two recently approved therapies, ceftolozane-tazobactam and ceftazidime-avibactam.

**Methods:**

This systematic literature review surveys the published clinical trial evidence available since 2000 in support of both current and emerging treatment options in the settings of complicated intra-abdominal infection (cIAI) and complicated urinary tract infection (cUTI). When available, clinical cure rates for patients with infections from ESBL-producing strains are provided, as is information about efficacy against *Pseudomonas aeruginosa*.

**Results:**

Clinical trial evidence to guide selection of empiric antibiotic therapy in patients with complicated, hospital-acquired, Gram-negative IAIs and UTIs is limited. Though most of the clinical trials explored in this overview enrolled patients with complicated infections, often patients with severe infections and multiple comorbidities were excluded.

**Conclusions:**

Practitioners in the clinical setting who are treating patients with complicated, hospital-acquired, Gram-negative IAIs and UTIs need to consider the possibility of polymicrobial infections, antibiotic-resistant organisms, and/or severely ill patients with multiple comorbidities. There is a severe shortage of evidence-based research to guide the selection of empiric antibiotic therapy for many patients in this setting. New therapies recently approved or in late-stage development promise to expand the number of options available for empiric therapy of these hospital-acquired, Gram-negative infections.

## Background

The increasing prevalence of bacterial infections with resistance to currently available antibiotics and the limited number of new antibiotics in development are now well-documented [1, 2]. This issue is especially acute for Gram-negative, healthcare-associated infections (HAIs), prompting the U.S. Centers for Disease Control and Prevention (CDC) to issue warnings regarding Gram-negative organisms, highlighting both the ability of these organisms to develop drug resistance and the scarcity of new treatments to combat them [3].

Antibiotic-resistant Gram-negative infections are especially prevalent in HAIs, accounting for about one-quarter to one-third of such infections overall [4]. Gram-negative pathogens are frequently isolated from healthcare-acquired intra-abdominal infections (IAI) and urinary tract infections (UTIs) [4, 5]. β-Lactam antibiotics are the traditional antibiotic class for infections caused by Gram-negative bacteria [3]. The highly-adaptive Gram-negative pathogens can produce various β-lactamase enzymes that render them resistant to the antibiotic’s mechanism of action. Extended-spectrum β-lactamase (ESBL)-producing Gram-negative bacteria also tend to harbor resistance to several classes of non–β-lactam antibiotics, including fluoroquinolones, aminoglycosides, and trimethoprim-sulfamethoxazole [6]. As a consequence, these classes of antibiotics are generally associated with worse outcomes when treatment needs to account for the possibility of an ESBL-producing strain [7–10]. Carbapenems are generally recommended as first-line empiric therapy for Gram-negative infections in this case, and β-lactam/β-lactamase–inhibitor combinations such as piperacillin-tazobactam are a second-line option [8, 10]. However, the strength of evidence supporting these recommendations is variable. Recent clinical trials have supported the use of novel or third-generation cephalosporins in combination with a β-lactamase inhibitor in this setting.

Empiric therapy for Gram-negative HAIs needs to take into account local susceptibility data [10] as well as the risk for the presence of variousdrug-resistant strains. Of particular concern are ESBL-producing and carbapenemase-producing Enterobacteriaceae, as well as multi-drug–resistant *Acinetobacter*, and *Pseudomonas aeruginosa* [3, 6, 8]. Empiric therapy must balance the potential benefits of appropriate therapy with the potential for selection of resistant strains. The purpose of this review is to provide an overview of the clinical trial evidence supporting these established and emerging options for empiric therapy in patients with healthcare-associated complicated intra-abdominal infections [cIAIs] or healthcare-associated complicated urinary tract infections [cUTIs] in which treatment must account for the risk of ESBL-producing Gram-negative pathogens and other multi-drug–resistant Gram-negative strains as well as *P. aeruginosa*.

### Epidemiology

In the United States, ESBL-producing and carbapenem-resistant Enterobacteriaceae account for about 35,000 HAIs and 2300 deaths each year [3]. Between 2000 and 2009, the percentage of UTI infections from *Escherichia coli* and *Klebsiella pneumoniae* exhibiting ESBL production more than doubled (from 3.3 % to 8.0 % for *E. coli* and from 9.1 % to 18.6 % for *K. pneumoniae*) [11]. Overall, the frequency of UTI hospitalizations in the United States caused by resistant, Gram-negative pathogens increased by about 50 % for multi-drug–resistant *P. aeruginosa* and by about 300 % for ESBL-producing organisms [11]. The percentage of Enterobacteriaceae isolates from U.S. institutions exhibiting carbapenem resistance increased from 1.2 % in 2001 to 4.2 % in 2011, with *Klebsiella* species accounting for most of the increase [12]. Despite the relatively low incidence of infections from carbapenem-resistant *K. pneumoniae,* they are associated with high morbidity, mortality, and utilization of healthcare resources [13].

## Methods

**Fig. 1:**
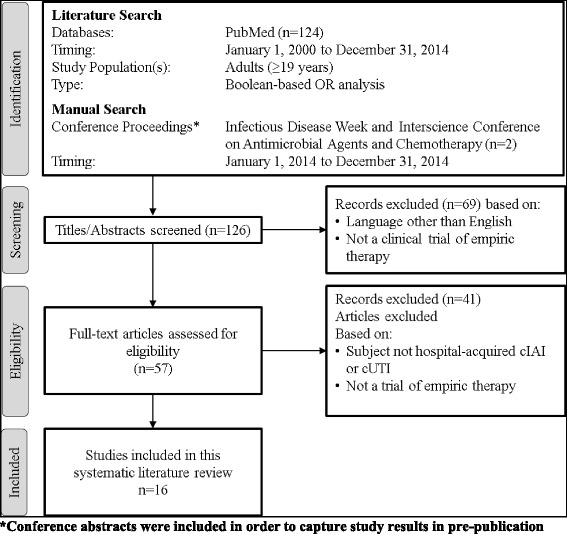
Flow diagram of literature search and study selection

Literature searches were performed on PubMed using generic drug names (and alternative names) as primary search terms (Table 1). Results were filtered to include only phase 2, 3, and 4 clinical trials. In most cases, results were limited to trials published from 2000 to present. Publications were manually selected to include only trials of empiric therapy in adult patients with hospital-acquired cIAI or cUTI. In addition, to capture recent studies in pre-publication, abstracts from 2014 Infectious Disease Week (IDWeek) and the Interscience Conference on Antimicrobial Agents and Chemotherapy (ICAAC) were manually searched using the same criteria as above. Other studies known to the author were also included. Individual case study reports were not included (Fig. 1).

**Table 1:** Criteria for scientific literature search related to current and emerging treatment options for hospital-acquired, gram-negative complicated intra-abdominal infection and complicated urinary tract infections

• Literature Databases
- US National Library of Medicine National Institutes of Health; PubMed; PubMedCentral; Medline
• Search Terms
- avibactam; carbapenem; ceftazidime; ceftolozane; ceftolozane tazobactam plus metronidazole; cephalosporin; cilastatin; doripenem; imipenem; meropenem; moxifloxacin; tazobactam; tigecycline; complicated urinary tract infection; complicated intra-abdominal infection
• MeSH Headings
- All
• Search Type
- Boolean-based OR analysis
• Study Population(s)
- Adults (≥19 years)
• Species
- Human
• Language(s)
- English
• Article Types
- Clinical trial; Clinical trial phase II; Clinical trial phase III; Clinical trial phase IV
• Journal Categories
- All
• Timeframe
- January 1, 2008 to December 31, 2014

The systematic literature review yielded 16 clinical studies which were carried out during 2004 – 2014. A total of two clinical studies were identified via manual search of medical congress database presentations. All remaining clinical studies (*n* = 14) were published in peer-reviewedmedical journals. Of these 14 published clinical trials, 13 studies explicitly reported that written informed consent was obtained for each patient and the protocol was reviewed and approved by an ethical review committee. Financial support was disclosed for all studies: all had received funding from the company that manufactured the antibiotic. A statement related to potential conflicts of interest was reported for 10 of the studies.

High-quality studies were defined as phase 3, randomized, controlled, double-blind trials or pooled analyses of such trials. Medium-quality studies include any phase 3 trials that substantially deviate from the high-quality definition (such as open-label or single-arm trials) as well as phase 2 trials. Low-quality studies include all other studies meeting the search criteria. Whenever sufficient high-quality data were available, low-quality studies were not included. Data was extracted manually. Unless otherwise noted, clinical cure rates at the test-of-cure (CCR-TOC) endpoint are given for the intent-to-treat population. The tables showing trial results are not intended for comparison of cure rates across trials, owing to differences in patient demographics and clinical characteristics, variation within trial design, as well as differences in the prevalence of Gram-negative organisms across different institutions, regions, and time epochs. Due to the paucity of data, potential sources of bias – such as study sponsor, study design, study location, and number and type of study sites – were not considered in the design of this systematic literature review.

## Results

### Complicated intra-abdominal infection (cIAI)

Among the indications included in this overview, cIAI had the largest set of high- and medium-quality supporting trials. Key results are summarized in Table 2. Overall, use of the following therapies was supported by at least one high-quality trial: doripenem, meropenem, imipenem-cilastatin, ertapenem, tigecycline, piperacillin-tazobactam, andceftolozane-tazobactam. However, the phase 3 ceftolozane-tazobactam trial is not yet published in a peer-reviewed journal (as of February 2015). The newest option is ceftazidime-avibactam, a non–β-lactam β-lactamase inhibitor in combination with a late-generation cephalosporin, which is supported by medium-quality evidence from a phase 2 trial. In the high-quality trials, doripenem and ceftolozane-tazobactam provided the strongest evidence of efficacy against ESBL-producing strains and *P. aeruginosa*. Tigecycline was associated with significantly higher rates of nausea and vomiting vs. the comparator (imipenem-cilastatin). In one high-quality trial from 2003 (Study G, Table 1), ertapenem was associated with clinical cure in 73.1 % (19/26) of patients with *P. aeruginosa,* although baseline isolates showed only 60 % susceptibility [14]. In general, ertapenem does not have reliable efficacy against *P. aeruginosa* (see Discussion).

**Table 2:** Summary of studies in complicated intra-abdominal infection (cIAI)

		CCR-TOC^a^					
Index	Reference	Agent A	Agent B	Description	Quality high (H) or medium (M)	Comparative outcome (A vs B)	Year
A	[27]	Doripenem mITT 85.9 % (140/163)	Meropenem mITT 85.3 % (133/156)	R, DB, P3	H	Noninferior	2008
B	[28]	Tigecycline 86.1 % (441/512)	Imipenem-cilastatin 86.2 % (442/513)	Pooled analysis of two phase 3 trials.	H	Noninferior	2005
C	[29]	Tigecycline 92.4 % (219/237)	Imipenem-cilastatin 88.8 % (198/223)	Subanalysis of the European data from Study B	H	-	2008
D	[30]	Tigecycline 80.6 % (199/247)	Imipenem-cilastatin 82.4 % (210/255)	R, DB, P3	H	-	2005
E	[31]	Tigecycline mITT 86.5 % (45/52)	Imipenem-cilastatin mITT 97.9 % (47/48)	R, OL, P3	M	-	2010
F	[32]	Tigecycline 81.8 % (162/198)	Ceftriaxone-metronidazole 79.4 % (150/189)	R, OL	M	Noninferior	2012
G	[14]	Ertapenem 79.3 % (245/311)	Piperacillin-tazobactam 76.2 % (232/304)	R, DB, P3	H	Equivalent	2003
H	[33]	Piperacillin-tazobactam “Clinical success” 97.3 % (108/111)	Imipenem-cilastatin “Clinical success” 97.1 % (100/103)	R	M/H	-	2004
I	[34]	Piperacillin-tazobactam followed by amoxicillin clavulanate For HAI: 55 % (17/31)	Moxifloxacin For HAI: 82 % (22/27)	R, DB, P3	M		2006
J	[35]	Ceftolozane-tazobactam + metronidazole 91.4 % (64/70)	Meropenem 94.3 % (33/35)	R, DB, P2	M	-	2014
K	[36]	Ceftolozane-tazobactam 83.8 % (399/476)	Meropenem 85.8 % (424/494)	R, DB, P3	H	Noninferior	
L	[37]	Ceftazidime-avibactam + metronidazole 91.2 % (62/68)	Meropenem 93.4 % (71/76)	R, P2	M	-	2013

*CCR-TOC* clinical cure rate at the test-of-cure endpoint, *DB* double-blind, *HAI* hospital-acquired infection, *MIC* minimum inhibitory concentration, *mITT* microbiologically evaluable intent-to-treat population, *OL* open-label, *P2* phase 2, *P3* phase 3, *R* randomized.

^a^Unless otherwise noted.

### Complicated urinary tract infection (cUTI)

Only three high-quality and one medium-quality studies were found for treatment of cUTI (Table 3). High-quality trials support the use of doripenem (or levofloxacin). Two studies (three trials) found high clinical cure rates for doripenem, including activity in patients with levofloxacin-resistant *E. coli*. Ceftolozane-tazobactam and levofloxacin had high clinical cure rates overall, but levofloxacin had low rates of microbiological eradication in patients with ESBL-producing Enterobacteriaceae or *P. aeruginosa*. A phase 2 trial supports the use of ceftazidime-avibactam plus metronidazole or imipenem-cilastatin.

**Table 3:** Summary of studies in complicated urinary tract infection (cUTI)

		CCR-TOC/ITT^a^					
Index	Reference	Agent A	Agent B	Description	Quality	Comparative outcome (A vs B)	Year
M	[38]	Doripenem 95.1 % (272/286)	Levofloxacin 90.2 % (240/266)	P3, R	H	Noninferior	2009
N	[39]	Doripenem 94.1 % (511/543)	Levofloxacin 90.2 % (240/266)	See comments	H	Noninferior	2010
O	[40]	Ceftolozane-tazobactam 95.9 % (327/341) ME	Levofloxacin 93.2 % (329/353) ME	R, DB, P3	H	Noninferior	
P	[41]	Ceftazidime-avibactam 85.7 % (24/28)	Imipenem-cilastatin 80.6 % (29/36)	P2, DB, R	M	-	2012

*CCR-TOC/ITT* clinical cure rate at the test-of-cure endpoint intent-to-treat, *DB* double-blind, *ME* microbiological eradication, *OL* open-label, *P2* phase 2, *P3* phase 3, *R* randomized.

^a^Unless otherwise noted.

## Discussion

The overarching finding from this overview is that there is only sparse clinical trial evidence (since the year 2000) to guide selection of empiric antibiotic therapy in patients with complicated, hospital-acquired, Gram-negative IAIs and UTIs. Given that practitioners working in these settings frequently need to take into account the potential for polymicrobial infections, ESBL-producing organisms, and multi-drug–resistant *Pseudomonas* and other species, the evidence supporting therapy selection appears even less satisfactory. The inadequacy of the available evidence is further highlighted when severity of illness is considered. In the setting of hospital-acquired microbial infection, patients are often severely ill with multiple comorbidities. Most of the clinical trials explored in this overview, even though they enrolled patients with complicated infections, excluded patients with APACHE (Acute Physiology and Chronic Health Evaluation) II scores >30. Indeed, most of the trials had median APACHE II scores of 5–7, and patients were excluded on the basis of many common comorbid conditions. Thus, there is a severe shortage of evidence-based guidance on empirical antibiotic therapy for many patients with hospital-acquired, Gram-negative infections.

Considering the available evidence, the carbapenems in general—and doripenem in particular—are associated with the strongest set of high-quality trials. Doripenem, meropenem, imipenem-cilastatin, and ertapenem all had support from high-quality trials in the setting of cIAI, and doripenem also had support for use in cUTI. Carbapenems (sometimes in combination with another antibiotic) are active against most ESBL-producing strains and have been recommended as first-line therapy in critically ill patients with severe infections when there is a risk of ESBL-producing, Gram-negative bacteria [1, 8, 10, 15]. However, they are susceptible to carbapenemases. Imipenem, meropenem, and doripenem are active against *P. aeruginosa,* although doripenem has a lower MIC and was superior to imipenem in that regard in patients with cIAI [16]. Ertapenem is not reliably active against *P. aeruginosa* [16]. Doripenem is less susceptible to certain carbapenemases compared with other carbapenems, although it is still susceptible to metallo-β-lactamase (MBL)-producing strains [17].

Tigecycline has broad-spectrum activity against both Gram-positive and Gram-negative bacteria, including activity against ESBL-producing strains. Its use in cIAI was associated with strong supporting evidence, although it was associated with significantly higher rates of nausea and vomiting. One medium-quality trial of tigecycline found high rates of clinical cure in patients with cIAI due to *P. aeruginosa* (Study F), but tigecycline is not reliably active against *P. aeruginosa* and its activity against *Providencia* and *Proteus* strains can be limited [18]. It has been widely studied for skin and skin-structure infections (not shown here) but has limited penetration into the urinary tract [19, 20]; thus, there are limited data about its use for treatment of UTI. Current data do not support its use in severe infections. In a meta-analysis, tigecycline was associated with significantly higher rates of adverse events and numerically higher mortality than comparators [21]. U.S. prescribing information for tigecycline contains a black-box warning about the increased mortality and indicating that its use should be reserved for situations when other treatments are not suitable.

Piperacillin in combination with the β-lactamase inhibitor tazobactam is another treatment option; however, its use is mostly limited to mild infections or UTI. Most of the evidence supporting piperacillin-tazobactam comes from small trials, except for one phase 3 trial in cIAI in which it was used as a comparator against ertapenem. Even though piperacillin-tazobactam was associated with high rates of clinical response in patients with *P. aeruginosa* in that trial (Study G), additional coverage using aminoglycosides or colistin is usually considered necessary if *P. aeruginosa* is a potential pathogen [10].

The two newest antibiotics targeting treatment of suspected ESBL-producing, Gram-negative infections are ceftolozane-tazobactam and ceftazidime-avibactam [22, 23]. Ceftolozane is a novel cephalosporin with improved stability against AmpC β-lactamases [22], although coverage of AmpC-containing species is not uniform [24]. It has a lower MIC for *P. aeruginosa* compared with other third-generation cephalosporins and its increased affinity to penicillin-binding proteins is thought to confer greater activity against resistant strains with efflux pumps or loss of porin channels [22]. Its use in combination with tazobactam confers greater activity against ESBL-producing Enterobacteriaceae [22]. However, it is not active against KPC-producing or MBL-producing strains. The two trials of ceftolozane-tazobactam (Studies J and O), to date available only in preliminary form, provide high-quality support for use of this therapy in patients with cIAI and cUTI.

Ceftazidime in combination with the non-β-lactam β-lactamase inhibitor avibactam is active against strains producing KPC or OXA-48 β-lactamases, as well as strains with carbapenem resistance owing to porin loss and production of an ESBL or AmpC [25, 26]. *In vitro* studies found MIC ≤2 mcg/mL for ceftazidime-avibactam against 8 of 8 ceftazidime-resistant *K. pneumoniae* isolates with OXA-48, 15 of 15 isolates with combinations of impermeability and ESBLs or AmpC, and 7 of 10 isolates with KPC [25]. Avibactam inhibits Ambler class A and C β-lactamases and some class D β-lactamases. Medium-quality trials support the efficacy for ceftazidime-avibactam in cIAI and cUTI, and phase 3 trials are ongoing.

## Conclusions

Clinical trial evidence is sparse regarding empiric antibiotic therapies for patients with hospital-acquired infections, when antibiotic therapy often needs to cover ESBL-producing strains, *P. aeruginosa*, and other multi-drug–resistant strains. Furthermore, the available clinical trial evidence is often incompatible with the clinical setting, in which patients have more severe illness or comorbidities excluded from clinical trials. Comparatively strong evidence exists for treatments in the setting of cIAI but the evidence in the setting of cUTI is small. When antibiotic therapy is necessary, current guidelines recommend empiric therapy using a carbapenem until definitive therapy can be selected. The limited clinical trial evidence supports this recommendation, although carbapenems differ in their coverage of *P. aeruginosa* and susceptibility to carbapenemases. Guidelines also recommend that choice of empiric therapy be tailored to account for knowledge of institutional and local susceptibility patterns. Unfortunately, clinical trials offer only scant information to aid in that aspect of decision making, leaving clinicians to rely on *in vitro* susceptibility testing.

## Abbreviations

APACHE: Acute Physiology and Chronic Health Evaluation
CCR-TOC: Clinical cure rates at the test-of-cure
CDC: Centers for Disease Control and Prevention
cIAI: Complicated intra-abdominal infection
cUTI: Complicated urinary tract infection
DB: Double-blind
ESBL: Extended-spectrum β-lactamase
HAI: Healthcare-associated infection
IAI: Intra-abdominal infection
ICAAC: Interscience Conference on Antimicrobial Agents and Chemotherapy
IDWeek: Infectious Disease Week
ITT: Intent to treat
KPC: *Klebsiella pneumoniae* carbapenemase
MBL: Metallo-β-lactamase
MIC: Minimum inhibitory concentration
ME: Microbiological eradication
mITT: Microbiologically evaluable intent-to-treat population
OL: Open-label
UTI: Urinary tract infection
